# High levels of vascular cell adhesion molecule-1 associate with a ‘vasculopathic’ phenotype in systemic sclerosis with higher mortality

**DOI:** 10.1093/rheumatology/keag391

**Published:** 2026-07-29

**Authors:** Matthew J S Parker, Mandana Nikpour, Dylan Hansen, Aimee L Hanson, Joanne Sahhar, Matthew A Brown, Tamera J Corte, Susanna Proudman, Tony J Kenna, Australian Scleroderma Interest Group

**Affiliations:** Faculty of Medicine and Health, The University of Sydney, Sydney, Australia; Department of Rheumatology, Royal Prince Alfred Hospital, Sydney, Australia; The Institute for Academic Medicine, Royal Prince Alfred Hospital, Sydney, Australia; Department of Rheumatology, Royal Prince Alfred Hospital, Sydney, Australia; The School of Public Health, University of Sydney, Sydney, Australia; Department of Rheumatology, St Vincent’s Hospital, Melbourne, Australia; MRC Integrative Epidemiology Unit, Bristol Medical School, University of Bristol, Bristol, UK; Department of Rheumatology, Monash Health, Melbourne, Australia; Department of Medicine, Central Clinical School, Monash University, Melbourne, Australia; Department of Medical and Molecular Genetics, King’s College London, London, UK; NHMRC Centre of Research Excellence in Pulmonary Fibrosis, Sydney, Australia; Department of Respiratory Medicine, Royal Prince Alfred Hospital, Sydney, Australia; Rheumatology Unit, Royal Adelaide Hospital, Adelaide, Australia; Discipline of Medicine, University of Adelaide, Adelaide, Australia; Centre for Immunology and Infection Control, School of Biomedical Sciences, Queensland University of Technology, Brisbane, Australia

**Keywords:** scleroderma, scleroderma heart disease, digital ulceration, biomarker, prognosis

## Abstract

**Objectives:**

To determine disease-specific associations of serum vascular cell adhesion molecule-1 (VCAM-1) and associated mortality in SSc.

**Methods:**

Participants were identified from the Australian Scleroderma Cohort Study. Data were linked with the National Death Index for cause-specific mortality. VCAM-1 was measured using a magnetic Luminex assay. Participant characteristics and information on organ specific manifestations were extracted until February 2024. Participants were stratified into VCAM-1 quartiles.

**Results:**

Of 388 participants, 87.1% were female and 76.8% had limited cutaneous disease. Median age at diagnosis was 45.7 years (interquartile range 36.4–56.7). Participants with upper quartile VCAM-1 (quartile 4; Q4) had increased mortality compared with others [hazard ratio (HR) 2.17, 95% CI 1.54–3.04; *P* < 0.001]. Despite the significant increased mortality in Q4, there were no statistically significant differences in sex, age, disease duration, disease subtype, autoantibody profile or forced vital capacity across the VCAM-1 quartiles. Q4 were more likely to have pulmonary arterial hypertension (PAH; *P* = 0.028), SSc-attributable myocardial disease (*P* = 0.009) and digital ulcers (*P* = 0.003). In cause-specific mortality analysis, Q4 were more likely to have PAH (HR 3.08, 95% CI 1.68–5.65; *P* < 0.001), SSc-attributable myocardial disease (HR 2.85, 95% CI 1.51–5.38; *P* = 0.001) and all-cause cardiovascular disease (HR 2.50, 95% CI 1.60–3.89; *P* < 0.001) listed as a cause or contributor to death. Q4 VCAM-1 level was not associated with interstitial lung disease presence, severity or cause-specific mortality.

**Conclusion:**

The increased mortality in participants with SSc and Q4 VCAM-1 levels is attributable to increased frequency of vascular disease manifestations. Q4 participants do not have a disproportionate frequency of other established risk factors for increased mortality, suggesting an independent role for VCAM-1 in disease pathophysiology.

Rheumatology key messages

- High serum levels of vascular cell adhesion molecule-1 (VCAM-1) are associated with increased mortality in SSc.
- The increased mortality in participants with high VCAM-1 levels is attributable to increased frequency of vascular disease manifestations.
- The association with mortality is independent of previously established prognostic factors.

## Introduction

SSc is an autoimmune disease characterized by fibrosis and vasculopathy [1]. Despite advances in pathogenesis and management, population-level mortality has changed little over 40 years [2, 3]. There is increasing evidence that contemporary management approaches such as regular screening for early organ complications, multimodal therapy interventions such as the combination of MMF and antifibrotics in interstitial lung disease (ILD), the use of dual or triple vasodilator therapy in pulmonary arterial hypertension (PAH), or the use of autologous stem cell transplant and cellular therapies in carefully selected patients, can improve outcomes for individual patients [4–7]. The potentially effective therapeutic options often come with significant potential toxicity, for example the >5% early mortality rate in autologous stem cell transplant, and it remains challenging for clinicians to determine which patients should be offered treatment [8]. It is well recognized that not all patients with SSc progress to serious organ involvement and some do not progress even once they have serious organ involvement.

Much work has been done to better understand which patients with SSc are likely to have a higher mortality risk. Established risk factors for increased mortality include the presence of serious SSc-related organ complications such as PAH and ILD, certain patient factors such as male sex, older age and smoking status, as well as particular disease characteristics such as diffuse disease, rapidly progressive skin disease and the anti-Scl70 (topoisomerase I) antibody [9, 10]. These risk factors for mortality and disease progression incompletely account for the diversity in patient outcomes and accurate prognostication remains an unmet clinical need.

Serum biomarkers are showing promise for improving prognosis, for example the use of N-terminal pro-B-type natriuretic peptide (NT-proBNP) for screening for the presence of PAH in SSc [11]. In prior work, our group investigated the performance of 27 serum biomarkers, the selection of which was informed by a prior literature review [12, 13]. Amongst numerous potentially important results, the most striking was the strong association between mortality and high serum levels of vascular cell adhesion molecule-1 (VCAM-1), with a hazard ratio (HR) of 3.55 (95% CI 2.37–5.33; *P* < 0.001). The strong association remained when adjusting for the established risk factors detailed above, suggesting that serum VCAM-1 levels may help to further refine prognostication in SSc.

If the previously novel strong association between VCAM-1 and SSc mortality were replicated, the main aim of this study was to determine disease-specific associations of serum VCAM-1 and associated mortality in SSc.

## Patients and methods

We included patients with SSc in the Australian Scleroderma Cohort Study (ASCS) who had stored serum available. The ASCS is a national, multicentre, longitudinal observational cohort established in 2007 by the Australian Scleroderma Interest Group. Patients fulfilling ACR/EULAR classification criteria for SSc are eligible for enrolment [14]. Participants provided written consent, and de-identified clinical, treatment and outcome data are collected at baseline and annually. Serum is stored at baseline and at annual visits. ASCS has human research ethics approval from all participating centres.

### Definitions

Baseline was the first serum collection used for VCAM-1 measurement. Disease duration was calculated from first non-Raynaud symptom until baseline, and disease subgroup was classified as limited or diffuse by the enrolling physician based on the extent of peak skin involvement according to Leroy criteria [15]. Definitions of organ manifestations mirror internationally accepted definitions, where established, and are only recorded when considered directly attributable to SSc. Organ manifestations were recorded as ever present, present before baseline or incident after baseline. ILD is defined by presence on high-resolution CT (HRCT) chest. ILD extent was defined with ‘limited disease’ considered <20% involvement on HRCT and ‘extensive disease’ as >20% involvement [16]. PAH was defined using right heart catheter measured haemodynamic criteria according to the 2019 European Society of Cardiology/European Respiratory Society criteria (mean pulmonary artery pressure ≥20 mmHg, pulmonary artery wedge pressure ≤15 mmHg and peripheral vascular resistance ≥3 WU) [17]. In a small proportion of participants where peripheral vascular resistance was not recorded, the pre-existing definition of PAH (mean pulmonary artery pressure ≥25 mmHg) was applied [18]. Echocardiography-derived haemodynamics are presented as systolic pulmonary artery pressure and as mean pulmonary artery pressure, the latter calculated as per Chemla *et al*. [19] Percentage predicted values for forced vital capacity (FVC%) were reported using the Global Lung Initiative 2012 predictive equation [20]. In this cohort, SSc-attributable myocardial disease (SSc-myocardial disease) is clinician defined, informed by relevant investigations including serum cardiac enzymes, echocardiography and cardiac MRI where available. Cardiac investigations are organized as felt clinically indicated. SSc-myocardial disease is only recorded where the clinician is of the opinion that the cardiac disease is not attributable to an alternative aetiology.

The presence and persistence of comorbidities such as systemic hypertension, ischaemic heart disease and peripheral (arterial) vascular disease are recorded at enrolment and each annual review. Prior malignancy excludes non-melanoma skin cancers. Medications included in analyses are those recorded at or in the review immediately prior to baseline. Baseline serum CRP, ESR and uric acid, or the most recent recorded result within 12 months were used where available.

Mortality is reported as all-cause mortality unless otherwise specified. Cause-specific mortality was derived through linkage to the National Death Index which records primary and any contributing causes of death; linkage data were current to March 2023. International Classification of Diseases (ICD)-10 codes used for cause-specific mortality are included in Supplementary Table S1. PAH-contributed mortality was defined by ICD-10 codes, as either ICD I27.0 or ICD I27.2. SSc-myocardial disease contributed mortality was defined by ICD-10 codes I3*, I4*, I50*, I51* or I53*. All-cause cardiovascular mortality was defined as I*, R57.0*, R57.1* or R58*. ILD-contributed mortality was defined as J84. Follow-up data were censored on 29 February 2024.

### Serum samples and biomarker measurement

Serum samples were obtained from the ASCS biobank, stored at −80°C and excluded if they had undergone more than two freeze–thaw cycles. VCAM-1 were measured via magnetic Luminex according to manufacturer instructions. Serial samples were measured where available.

### Statistical analysis

Continuous variables are reported as mean (s.d.) or median (interquartile range), and categorical variables as number (%). A *P*-value <0.05 was considered significant.

VCAM-1 was analysed as a continuous variable and by quartile for a broad range of relevant outcomes. Group comparisons used chi-squared, analysis of variance, Kruskal–Wallis, *t* test or Wilcoxon rank-sum tests as appropriate. Univariable and multivariable analyses were performed, and survival was analysed using Cox proportional hazards regression. Analyses were performed in STATA 15.1 (StataCorp, College Station, TX, USA).

Analyses were performed in four stages: validation of the VCAM-1 mortality association in an independent cohort; assessment of cause-specific mortality; examination of associations with disease manifestations and established mortality risk factors; and evaluation of whether serial VCAM-1 change added prognostic value [12].

## Results

We identified 388 participants of whom the majority were female (87.1%) and had limited cutaneous disease (76.8%). Median age at diagnosis was 45.7 years (36.4–56.7) and median disease duration at first VCAM-1 measurement was 13.6 years (8.16–21.4). Baseline characteristics are shown in Table 1. One hundred and forty-one participants (36.3%) were also included in our previous biomarker study [12].

**Table 1:** Participant characteristics at baseline presented across VCAM-1 quartiles.

	VCAM-1 (ng/ml)				*P*-value
	Q4 (>537; *n* = 97)	Q3 (414.7–537; *n* = 97)	Q2 (317.3–414.7; *n* = 97)	Q1 (<317.3; *n* = 97)	
Sex—female	86 (88.7)	85 (87.6)	84 (86.6)	83 (85.6)	0.928
Age at diagnosis	48.70 (39.07–58.14)	47.69 (36.64–55.87)	41.76 (36.18–51.50)	44.84 (33.75–53.17)	0.063
Disease duration at baseline	15.22 (8.83–24.75)	13.16 (8.51–20.59)	13.92 (8.07–21.28)	11.98 (7.22–19.01)	0.211
Diffuse disease (*n* = 89)	20 (21.3)	20 (20.6)	20 (20.6)	29 (30.5)	0.286
Limited disease (*n* = 299)	74 (78.7)	77 (79.4)	77 (79.4)	66 (69.5)	
Ever smoker	35 (36.1)	50 (51.5)	43 (44.3)	54 (56.3)	0.029
Hypertension	56 (58.3)	42 (43.3)	49 (50.5)	45 (47.9)	0.204
IHD	17 (17.7)	12 (12.4)	9 (9.3)	9 (9.4)	0.239
PAD	3 (9.4)	3 (9.4)	0 (0.0)	0 (0.0)	0.207
BMI >25 kg/m^2^	16 (17.6)	20 (22.7)	14 (15.6)	16 (17.8)	0.364
Prior malignancy	18 (18.8)	11 (11.3)	13 (13.4)	10 (10.4)	0.328
Alive at censorship	42 (43.3)	59 (60.8)	70 (72.2)	69 (71.1)	<0.001
Centromere	49 (51.0)	47 (48.5)	48 (49.5)	36 (37.5)	0.220
Scl-70	11 (11.5)	13 (13.5)	11 (11.3)	15 (15.6)	0.789
RNA pol. III	11 (14.1)	13 (17.3)	12 (15.6)	5 (9.1)	0.599
RNP	7 (7.3)	3 (3.1)	5 (5.2)	5 (5.2)	0.639
FVC% predicted at baseline	96.00 (81.00–105.90)	100.00 (84.00–114.00)	100.00 (86.00–113.00)	99.00 (86.00–111.00)	0.203
mRSS	7.00 (5.00–10.00)	6.00 (3.00–10.00)	5.00 (2.00–10.00)	5.00 (3.00–11.00)	0.124
Antiplatelet	38 (39.2)	34 (35.1)	25 (25.8)	27 (27.8)	0.157
Anticoagulant	15 (15.5)	10 (10.3)	10 (10.3)	8 (8.2)	0.424
Antihypertensive	84 (86.6)	78 (80.4)	73 (75.3)	73 (75.3)	0.160
ERA	23 (23.7)	16 (16.5)	15 (15.5)	21 (21.6)	0.398
PDEI	15 (15.5)	4 (4.1)	6 (6.2)	4 (4.1)	0.006

Data are presented as *n* (%) or median (interquartile range). VCAM-1: vascular cell adhesion molecule-1; Q1–4: quartile 1–4; IHD: ischaemic heart disease; PAD: peripheral arterial disease; Scl-70: anti-topoisomerase I antibody; RNA pol. III: anti-RNA polymerase III antibody; FVC: forced vital capacity; mRSS: modified Rodnan skin score; ERA: endothelin receptor antagonist; PDEI: phosphodiesterase inhibitor.

### VCAM-1 and mortality

**Figure 1:**
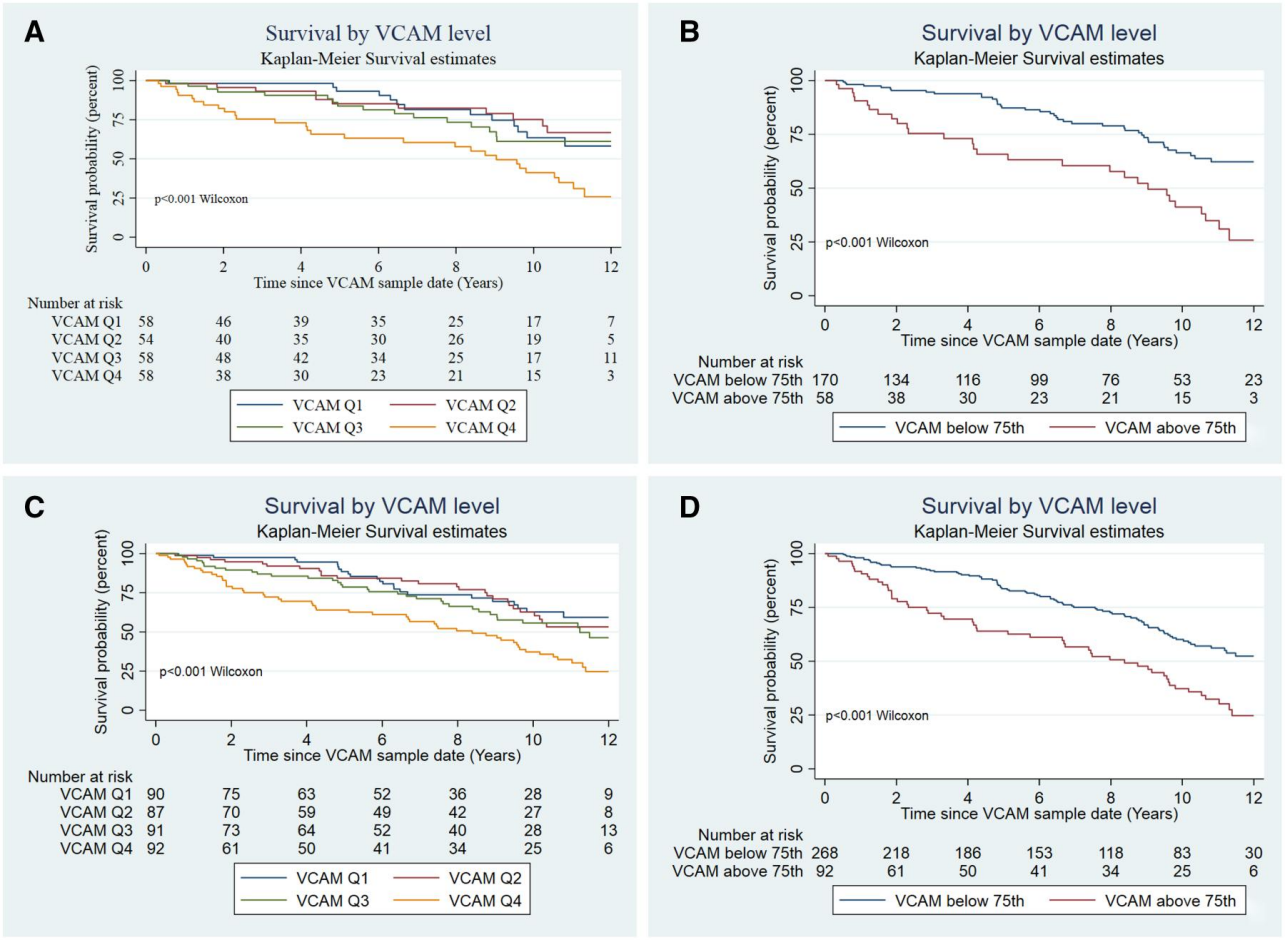
Kaplan–Meier survival curves. (A) Vascular cell adhesion molecule-1 (VCAM-1) quartiles in validation cohort (*n* = 247). (B) Quartile (Q) 1–3 *vs* Q4 in validation cohort (*n* = 247). (C) VCAM-1 quartiles in total cohort (*n* = 388). (D) Q1–3 *vs* Q4 in total cohort (*n* = 388)

In the pre-specified validation cohort (*n* = 247), higher VCAM-1 levels were associated with increased all-cause mortality. In univariable Cox analysis detailed in Fig. 1A and B, mortality was higher in participants with above mean levels of VCAM-1 (HR 1.74, 95% CI 1.09–2.78; *P* = 0.020) and highest in those in quartile 4 (Q4; HR 2.45, 95% CI 1.54–3.92; *P* < 0.001). The association remained significant after adjustment for age, sex, PAH and ILD (HR 1.81, 95% CI 1.07–3.06; *P* = 0.028).

Compared with participants included in the previously published derivation cohort, participants in the validation cohort (*n* = 247) were more often female (91.1% *vs* 80.8%, *P* < 0.001), had more limited disease (76.0% *vs* 66.9%, *P* = 0.013) and differed in autoantibody frequency (Supplementary Table S2). Because this study included substantial new analyses, subsequent results are presented for the full cohort (*n* = 388).

In the full cohort, median VCAM-1 was 414.7 ng/ml (interquartile range 317.3–537.0). Above mean VCAM-1 was associated with reduced survival (HR 1.72, 95% CI 1.23–2.41; *P* = 0.002) and participants in Q4 (VCAM1 > 537 ng/ml) had the highest mortality (HR 2.17, 95% CI 1.54–3.04; *P* < 0.001). This is detailed further in Fig. 1C and D.

Baseline characteristics are shown in Table 1. Despite higher mortality with increasing VCAM-1, quartiles did not differ significantly in sex, disease subtype, age, FVC%, disease duration, autoantibody profile, conventional cardiovascular risk factor or prior malignancy. This supports a more detailed analysis of the participants with the highest VCAM-1 levels in order to better understand why they may have increased mortality.

### Cause of death

**Figure 2:**
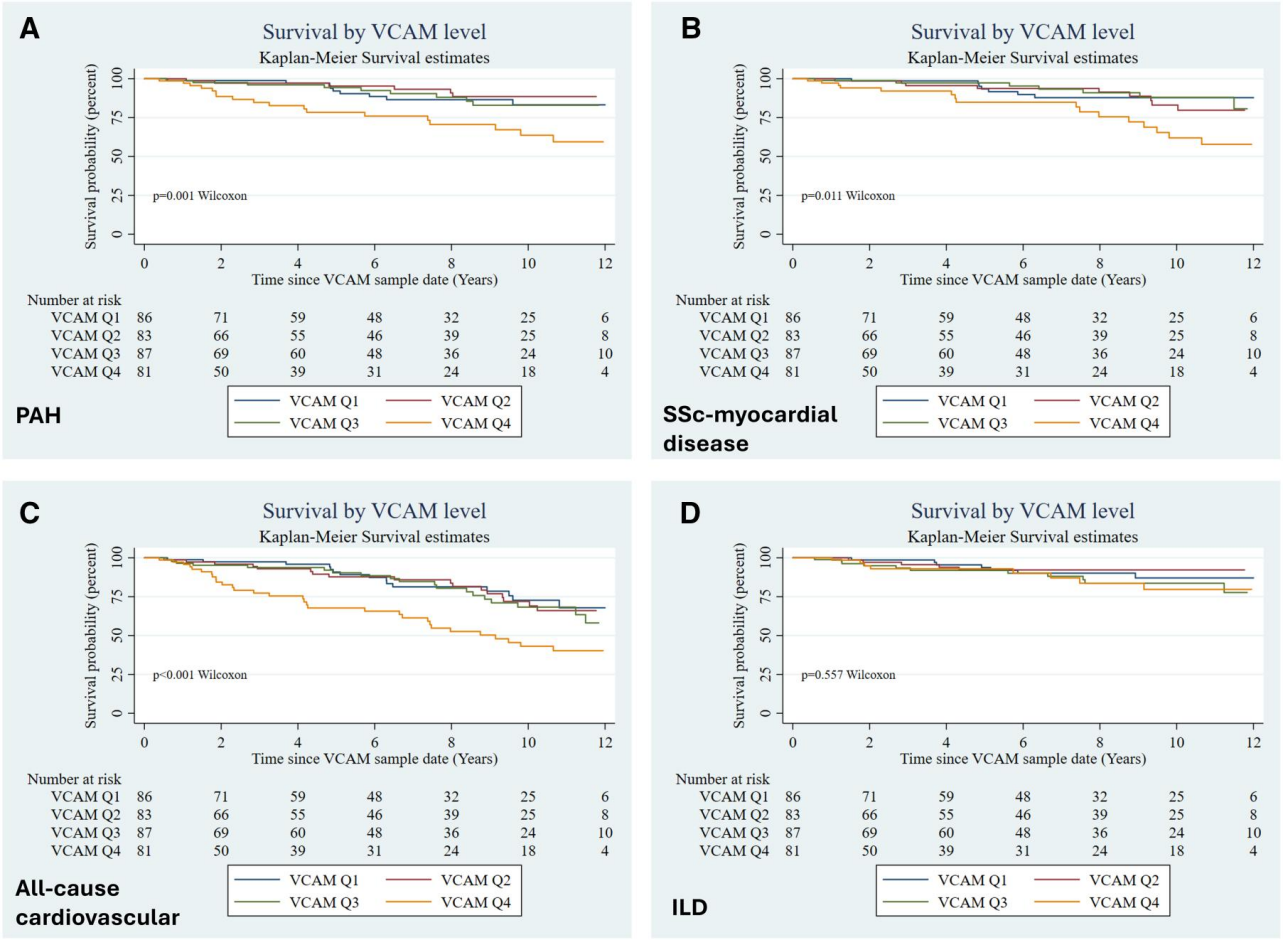
Kaplan–Meier survival curves from date of vascular cell adhesion molecule-1 (VCAM-1) sample collection by VCAM level quartiles. (A) Pulmonary arterial hypertension (PAH)-contributed mortality. (B) SSc-myocardial disease–contributed mortality. (C) All-cause cardiovascular disease–contributed mortality. (D) Interstitial lung disease (ILD)-contributed mortality. Note, not all patients have an International Classification of Diseases (ICD)-10 cause of death available; where no cause of death is available, the patient is excluded from survival analysis

Linked National Death Index data with ICD-10 codes were available for 343 participants (88.4%). Q4 VCAM-1 was associated with increased PAH-contributed mortality (HR 3.08, 95% CI 1.68–5.65; *P* < 0.001), SSc-myocardial disease–contributed mortality (HR 2.85, 95% CI 1.51–5.38; *P* = 0.001) and all-cause cardiovascular mortality (HR 2.50, 1.60–3.89; *P* < 0.001), but not ILD-related mortality (HR 0.49, 95% CI 0.06–3.89; *P* = 0.5). Figure 2 illustrates cause specific mortality in participants with Q4 VCAM-1 levels.

### Organ specific manifestations

Table 2 summarizes selected organ-specific manifestations, with additional analyses provided in Supplementary Table S3.

**Table 2:** SSc organ manifestations in Q4 *vs* Q1–3 participants.

SSc organ manifestation	Q4 VCAM-1 (*n* = 97)	Q1-3 VCAM-1 (*n* = 291)	*P*-value
RHC-defined PAH ever	16 (16.5)	29 (10.0)	0.082
RHC-defined PAH pre-baseline	**15 (15.5)**	**22 (7.6)**	**0.022**
RHC-defined PAH post-baseline	1 (1.5)	7 (3.1)	0.494
Echo sPAP, highest ever (mmHg)	**46.0 (37.5**–**58.5)**	**36.0 (31.0**–**46.0)**	**<0.001**
Echo sPAP (mmHg) pre-baseline	**41.0 (33.0**–**52.0)**	**34.0 (28.0**–**41.0)**	**<0.001**
Echo sPAP (mmHg) post-baseline	**44.0 (37.0**–**62.0)**	**35.0 (30.0**–**44.0)**	**<0.001**
Echo mPAP, Highest ever (mmHg)	**30.1 (24.9**–**37.7)**	**24.0 (20.9**–**30.1)**	**<0.001**
Echo mPAP (mmHg) pre-baseline	**27.0 (22.1**–**33.7)**	**22.7 (19.1**–**27.0)**	**<0.001**
Echo mPAP (mmHg) post-baseline	**28.8 (24.6**–**39.8)**	**23.4 (20.3**–**28.8)**	**<0.001**
NT-proBNP (pg/ml) pre-baseline	**221.3 (101.1**–**966.2)**	**101.7 (60.1**–**170.5)**	**<0.001**
Uric acid (mmol/L)	0.38 (0.25–0.43)	0.28 (0.24–0.34)	0.086
CRP (mg/L)	4.00 (3.50–5.80)	4.00 (2.00–5.00)	0.121
ESR (mm/h)	17.00 (9.00–27.00)	12.00 (8.00–23.00)	0.052
SSc-myocardial ever	**19 (19.6)**	**28 (9.6)**	**0.009**
SSc-myocardial pre-baseline	13 (14.1)	23 (8.2)	0.092
SSc myocardial post-baseline	**6 (9.7)**	**5 (2.2)**	**0.006**
HRCT-defined ILD ever	33 (34.0)	98 (33.7)	0.951
HRCT-defined ILD pre-baseline	27 (27.8)	80 (27.5)	0.948
HRCT-defined ILD post-baseline	6 (8.7)	18 (8.7)	0.991
Lowest FVC%	**83.5 (69.0**–**97.7)**	**90.0 (75.0**–**102.0)**	**0.035**
Lowest FVC % pre-baseline	**96.0 (81.0**–**105.9)**	**100.0 (85.0**–**113.0)**	**0.035**
Lowest FVC % post-baseline	84.3 (70.0–100.0)	91.4 (76.0–105.0)	0.071
Lowest DLCO %	**51.4 (39.2**–**65.7)**	**63.5 (47.4**–**75.7)**	**0.001**
Lowest DLCO % pre-baseline	**59.1 (44.8**–**71.7)**	**67.4 (54.8**–**80.1)**	**<0.001**
Lowest DLCO % post-baseline	**49.4 (38.7**–**63.3)**	**63.6 (46.9**–**75.2)**	**<0.001**
SSc renal crisis	**7 (7.3)**	**5 (1.7)**	**0.006**

Data are presented as *n* (%) or median (interquartile range). Statistically significant results are in bold type. VCAM-1: vascular cell adhesion molecule-1; Q1–4: quartile 1–4; Echo: echocardiogram; sPAP: systolic pulmonary artery pressure; mPAP: mean pulmonary artery pressure; NT-proBNP: N-terminal prohormone of brain natriuretic peptide; HRCT: high-resolution CT (chest); ILD: interstitial lung disease; FVC: forced vital capacity; DLCO: diffusing capacity for carbon monoxide; RHC: right heart catheter; VCAM-1: vascular cell adhesion molecule-1.

Q4 participants were more likely to have clinician-defined and haemodynamically defined PAH by baseline. Echocardiographic systolic pulmonary artery pressure (peroxidase–antiperoxidase) and mean peroxidase–antiperoxidase were higher, NT-proBNP levels were higher, and PAH-directed therapy was more common. Q4 participants also had lower diffusing capacity for carbon monoxide (DLCO%) before and after VCAM-1 measurement. In logistic regression, the odds ratio for right heart catheter–defined PAH by baseline in Q4 was 2.24 (1.11–4.51; *P* = 0.024).

Q4 participants also had a higher prevalence of clinician-defined SSc-attributable myocardial disease (19.6% *vs* 9.6%; *P* = 0.009), although this difference was only significant during follow-up.

Digital ulcers were more common prior to baseline in Q4 (62.5% *vs* 44.8%; *P* = 0.003). Scleroderma renal crisis was also more frequent by baseline in Q4 (7.3% *vs* 1.7%; *P* = 0.006).

There was no increased prevalence of clinician-defined ILD or HRCT-defined ILD in Q4. Although FVC% was lower at baseline, this was not explained by greater ILD or identified muscle involvement. There was no disproportionate distribution of gastro-oesophageal reflux, gastric antral vascular ectasia, myositis or calcinosis in Q4.

### Serial VCAM-1 measurements

**Figure 3:**
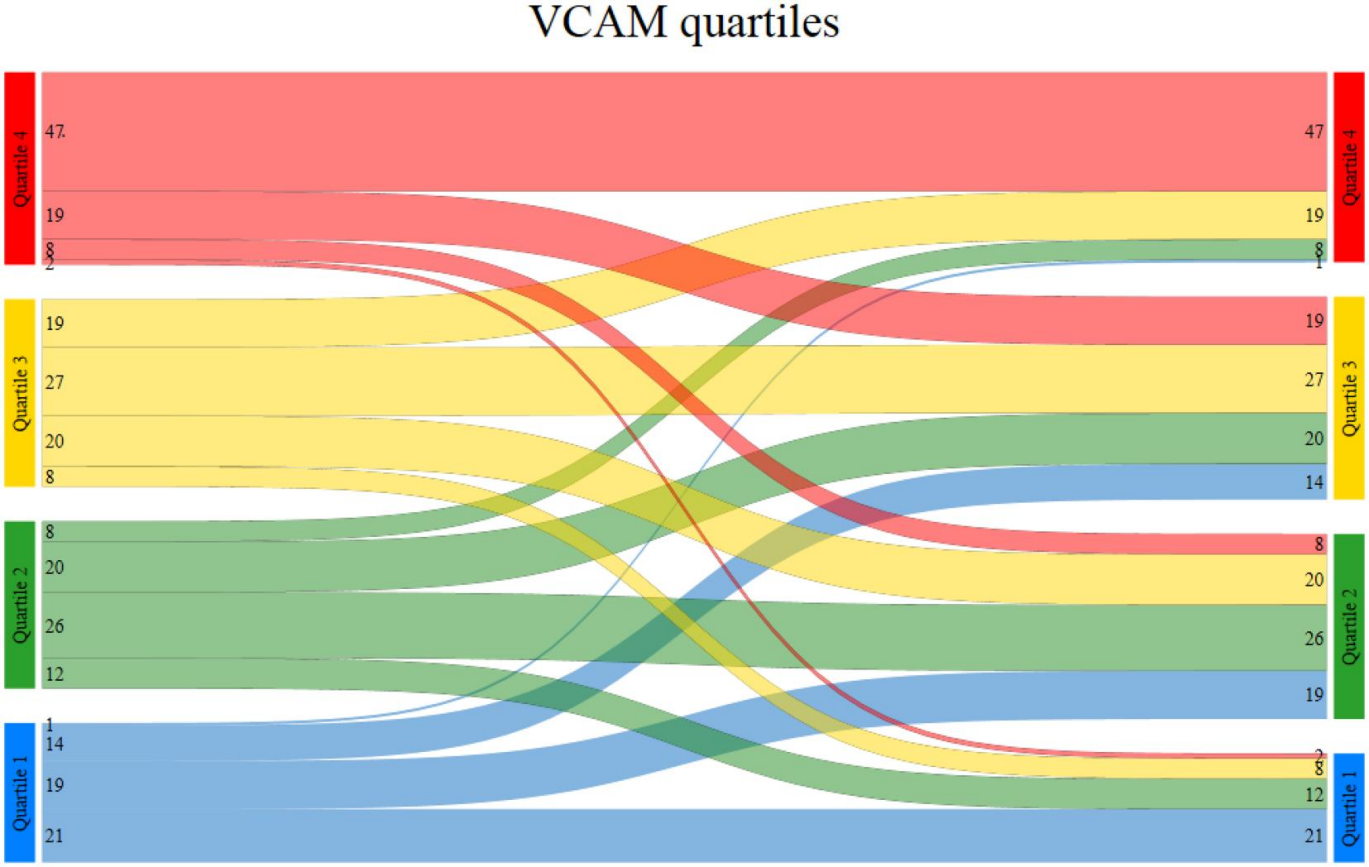
Sankey diagram of change in vascular cell adhesion molecule-1 (VCAM-1) quartiles from baseline to follow-up sample (quartile boundaries the same at both visits)

Serial VCAM-1 levels were measured in 271 participants over a median interval of 1.08 years (0.97–1.65). Change in VCAM-1, analysed continuously or by quartile movement, was not associated with disease manifestations or mortality, suggesting no prognostic value beyond the baseline level. Figure 3 shows quartile movement over time.

## Discussion

This study replicates our earlier finding that high VCAM-1 levels, particularly >537 ng/ml, are associated with high mortality in SSc [12]. Using National Death Index linkage, we further showed that participants in the highest VCAM-1 quartile had >3-fold higher PAH-related mortality and >2-fold higher SSc-myocardial and overall cardiovascular mortality.

We were able to examine the detailed phenotypes of participants stratified by VCAM-1 level with interesting results. Higher VCAM-1 was strongly associated with PAH, including clinician-defined and right heart catheter–defined disease, and with higher echocardiographic pulmonary pressures. This is biologically plausible because VCAM-1 is produced by endothelial cells in response to inflammatory stimuli and shear stress [21]. As most Q4 participants with PAH [21] already had PAH at measurement, VCAM-1 may reflect established endothelial activation rather than directly mediate disease. This may explain why our findings differ from those of Thakkar *et al.* [22] Their cohort was of a smaller size than that in this study (*n* = 15 SSc-PAH participants), and may not have been adequately powered to find a difference. It is also possible that there are truly different associations in different patient groups. The patients in this cohort have relatively long-established disease and studies of the relationship between VCAM-1 and PAH in prospective cohorts of patients with incident disease would be of interest. This study is not designed optimally to investigate whether vasoactive medications such as antiplatelets, anticoagulants, antihypertensives or PAH-directed therapies influence VCAM-1 levels and/or if this would have any clinical consequence, although these have been reported in our data and may be the focus of future work.

Participants with high VCAM-1 were more likely to have SSc-attributable cardiac disease, digital ulceration and scleroderma renal crisis. The increased frequency of myocardial disease is of particular relevance given evolving literature recognizing subclinical myocardial disease in a high proportion of patients who die with SSc [23]. Myocardial involvement is likely an under-diagnosed and under-appreciated manifestation of SSc [24]. A previous study has shown increased cardiovascular disease in SSc whilst controlling for traditional cardiovascular disease risk factors, suggesting SSc-specific factors involved in pathogenesis [25]. On the other hand, there is evidence from the cardiovascular literature that high VCAM-1 is associated with hypertension, atherosclerosis, ischaemic heart disease, stroke and heart failure [26]. In our dataset, the prevalence of conventional cardiovascular risk factors or manifestations was not statistically different across VCAM-1 quartiles. Whether the association between VCAM-1 and cardiovascular mortality is disease specific in SSc, more broadly relevant to the accelerated cardiovascular disease thought to account for much of the increased mortality attributable to CTDs or a combination of both, remains uncertain. Very few other biomarkers have been described to associate with SSc-heart disease. In this dataset, it appears that a high level of VCAM-1 may predict future myocardial disease rather than simply representing the presence of it. As the ASCS was established in 2007, the legacy term of ‘scleroderma myocardial involvement’ is used. However, this definition is consistent with the more recently published definition of ‘systemic sclerosis-associated primary heart involvement’ which requires direct attribution to SSc with other causes excluded [27].

Digital ulcers were more common in participants with the highest VCAM-1 levels. The presence of digital ulcers associates with other poor prognostic factors such as abnormal nailfold capillaroscopy, pulmonary hypertension and increased mortality [28–30] and is associated with significant morbidity in patients with SSc [31]. Although there are well developed tools for predicting digital ulcers and they are readily identifiable without the need for investigations, the association with digital ulcers, myocardial disease and pulmonary hypertension helps complete a triad of vascular (or ‘vasculopathic’) complications of SSc that are associated with high levels of VCAM-1. Some consider scleroderma renal crisis an additional vascular complication of SSc, a history of which was also substantially more common in Q4 participants. We postulate as a result that a high VCAM-1 level improves prognostication in patients with SSc because it serves as a serum biomarker of vasculopathic disease complications. This study has only suggested a possible link between vasculopathy and mortality in SSc. The study does not assess whether VCAM-1 levels are a specific biomarker for SSc-associated vasculopathy, or vascular disease in general. There may be value in studying VCAM-1 in other rheumatological diseases characterized by vasculopathy and/or higher cardiovascular mortality, and in relation to vascular disease and mortality in the general population.

In addition to PAH and cardiovascular disease, the other major cause of mortality in patients with SSc is ILD but there was no association between VCAM-1 and the presence of clinician-defined or HRCT-defined ILD or with ILD mortality. The association between high VCAM-1 and lower DLCO could be readily explained by PAH. The additional finding of a reduction in FVC% is less easy to explain but has been observed before in CTD-PAH [32]. It is possible that these patients could have other disease effects not captured accurately such as subclinical muscle involvement or truncal skin involvement [33]. VCAM-1 may be less relevant to fibrotic pathways than to vasculopathy. In a previous study we found other serum biomarkers such as SP-D were of greater prognostic potential in established SSc-ILD [12]. KL-6 has also been studied extensively and seems to predict the presence and progression of SSc-ILD [34].

Although VCAM-1 changed over time in some participants, serial measurement did not improve prognostication beyond the baseline level. The median interval between samples may have been too short to capture clinically meaningful progression.

Limitations include generalizability, as the cohort largely comprised patients with longstanding, mostly limited SSc, introducing potential selection and survivor bias. Annual assessments may affect accuracy of interpretation of incident manifestations, medication data may be incomplete and serum sampling was opportunistic rather than event based. ICD-10 cause-of-death coding is also imperfect in a rare multisystem disease. Another significant limitation of the present study is the absence of a defined normality threshold for VCAM-1 in healthy controls. Future studies inclusive of appropriately selected healthy controls will enable a better understanding of the potential clinical utility of VCAM-1 in patients with SSc.

Our data do not establish that VCAM-1 is prognostic of developing SSc organ manifestations or that it is causally linked to the various mortality outcomes but, in our opinion, do demonstrate a clear rationale for further work.

In conclusion, high baseline VCAM-1 was associated with the triad of serious vascular complications in SSc, namely pulmonary arterial hypertension, myocardial disease and digital ulceration. This study replicates and helps validate recent findings of VCAM-1 and high mortality in SSc. There is a strong case for further work to determine whether VCAM-1 can refine prognosis or illuminate vasculopathic mechanisms in SSc.

## Supplementary Material

keag391_Supplementary_Data

## Data Availability

Data will be shared upon request.
