# Supplementary material for: High levels of vascular cell adhesion molecule-1 associate with a ‘vasculopathic’ phenotype in systemic sclerosis with higher mortality
Source: Rheumatology (Oxford). 2026 Jul 29;65(8):keag391. doi: 10.1093/rheumatology/keag391 (PMC13485350; doi:10.1093/rheumatology/keag391)
Supplement: Supplementary file 1 [file keag391_supplementary_data.zip › rhe-25-1785-File002.docx]

## Supplementary Material:

**Supplementary Table S1: Cause-specific mortality definitions.** ICD 10, International Classification of Diseases 10th Revision; PAH, pulmonary arterial hypertension; SSc, Systemic sclerosis; ILD, Interstitial lung disease.

|  | **ICD 10 Codes** |
| --- | --- |
| PAH-contributed mortality | I27.0 OR ICD I27.2 |
| SSc-myocardial disease | I3*, I4*, I50*, I51* or I53* |
| All cause cardiovascular mortality | I*, R57.0*, R57.1*, or R58* |
| ILD-contributed mortality | J84 |

**Supplementary Table S2: Participant characteristics in validation cohort compared to derivation cohort.** (12) Statistically significant results in bold type. Baseline for each cohort may not be the same time (the studies were not conducted at the same time). Scl-70, anti-topoisomerase I antibody; RNA Pol. III, anti-RNA Polymerase III antibody; RNP, anti-ribonuclear protein antibody; RHC-defined PAH, right heart catheter-defined pulmonary arterial hypertension; SSc-myocardial, systemic sclerosis myocardial disease; HRCT-defined ILD, high resolution computated tomography (chest)-defined interstitial lung disease.

| Variable | Validation Cohort (n=247) | Derivation cohort (n=438) | p-value |
| --- | --- | --- | --- |
| Sex (Female) | **225 (91.1%)** | **354 (80.8%)** | **<0.001** |
| Age at diagnosis | 46.09 (36.82-54.65) | 46.70 (35.39-56.63) | 0.612 |
| Disease duration at baseline | 13.95 (8.01-21.17) | 13.44 (8.49-21.26) | 0.872 |
| Diffuse disease | **58 (24.0%)** | **145 (33.1%)** | **0.013** |
| Limited disease | **184 (76.0%)** | **293 (66.9%)** | **0.013** |
| Ever smoker | 113 (45.9%) | 222 (50.7%) | 0.233 |
| Alive at censorship | 170 (68.8%) | 289 (66.0%) | 0.447 |
| Centromere | **131 (53.5%)** | **136 (31.3%)** | **<0.001** |
| Scl-70 | **19 (7.8%)** | **115 (26.6%)** | **<0.001** |
| RNA pol. III | 26 (14.4%) | 51 (15.0%) | 0.855 |
| RNP | 14 (5.7%) | 18 (4.2%) | 0.358 |
| RHC-defined PAH ever | **20 (8.1%)** | **75 (17.1%)** | **0.001** |
| RHC-defined PAH pre-baseline | **19 (7.7%)** | **68 (15.5%)** | **0.003** |
| RHC-defined PAH post baseline | **1 (0.5%)** | **7 (6.5%)** | **0.003** |
| SSc myocardial ever | 30 (12.1%) | 47 (10.7%) | 0.573 |
| SSc myocardial pre-baseline | 22 (9.4%) | 44 (10.2%) | 0.740 |
| SSc myocardial post-baseline | 8 (4.4%) | 3 (2.7%) | 0.458 |
| HRCT-defined ILD ever | **41 (16.6%)** | **279 (63.7%)** | **<0.001** |
| HRCT-defined ILD pre-baseline | **28 (11.3%)** | **268 (61.2%)** | **<0.001** |
| HRCT-defined ILD post-baseline | **13 (6.0%)** | **11 (17.7%)** | **0.004** |

**Supplementary Table S3: Clinician-defined SSc organ manifestations, relevant comorbidities and treatments in Q4 *versus* Q1-3.** Significant differences in bold type. PAH, pulmonary arterial hypertension; ILD, interstitial lung disease; SSc myocardial, Systemic sclerosis myocardial disease; mRSS, modified Rodnan skin score; GAVE, Gastric antral vascular ectasia; IHD, ischaemic heart disease; PAD, peripheral arterial disease; ERA, endothelin receptor antagonist; PDEI, phosphodiesterase inhibitor.

|  | Q4 VCAM-1 (n=97) | Q1-3 VCAM-1 (n=291) | P-value |  |
| --- | --- | --- | --- | --- |
| PAH ever | **40 (41.2%)** | **85 (29.2%)** | **0.028** |  |
| PAH pre-baseline | **33 (34.4%)** | **67 (23.1%)** | **0.029** |  |
| PAH post-baseline | 7 (14.3%) | 18 (9.6%) | 0.345 |  |
| ILD ever | 35 (36.1%) | 103 (35.4%) | 0.903 |  |
| ILD pre-baseline | 31 (32.0%) | 84 (29.1%) | 0.590 |  |
| ILD post-baseline | 4 (7.7%) | 19 (10.8%) | 0.514 |  |
| SSc myocardial ever | **19 (19.6%)** | **28 (9.6%)** | **0.009** |  |
| SSc myocardial pre-baseline | 13 (14.1%) | 23 (8.2%) | 0.092 |  |
| SSc myocardial post-baseline | **6 (9.7%)** | **5 (2.2%)** | **0.006** |  |
| Myositis ever | 6 (6.2%) | 19 (6.5%) | 0.905 |  |
| Myositis pre-baseline | 5 (5.7%) | 17 (6.4%) | 0.835 | |
| Myositis post-baseline | 1 (1.5%) | 2 (0.9%) | 0.647 |  |
| Raynaud's ever | 94 (97.9%) | 270 (93.1%) | 0.078 |  |
| Raynaud's pre-baseline | 93 (96.9%) | 265 (91.7%) | 0.085 |  |
| Raynaud's post baseline | 1 (100.0%) | 5 (23.8%) | 0.095 |  |
| Digital ulcers ever | **60 (62.5%)** | **130 (44.8%)** | **0.003** |  |
| Digital ulcer pre-baseline | **50 (52.1%)** | **103 (35.5%)** | **0.004** |  |
| Digital ulcer post-baseline | 10 (29.4%) | 28 (18.5%) | 0.156 |  |
| mRSS | **7.00 (5.00-10.00)** | **5.00 (3.00-10.00)** | **0.043** |  |
| Reflux ever | 89 (92.7%) | 266 (91.7%) | 0.758 |  |
| Reflux pre-baseline | 89 (92.7%) | 252 (86.9%) | 0.124 |  |
| Reflux post-baseline | 0 (0.0%) | 14 (41.2%) | 0.073 |  |
| GAVE ever | 16 (16.5%) | 32 (11.0%) | 0.154 |  |
| GAVE pre-baseline | 14 (14.6%) | 24 (8.3%) | 0.072 |  |
| GAVE post baseline | 2 (3.2%) | 8 (3.5%) | 0.902 |  |
| Calcinosis ever | 57 (59.4%) | 162 (56.3%) | 0.592 |  |
| Calcinosis pre-baseline | 41 (42.7%) | 114 (39.6%) | 0.589 |  |
| Calcinosis post-baseline | 16 (39.0%) | 48 (33.6%) | 0.518 |  |
| Hypertension | 56 (58.3%) | 136 (47.2%) | 0.059 |  |
| IHD | 17 (17.7%) | 30 (10.3%) | 0.056 |  |
| PAD | 3 (9.4%) | 3 (3.8%) | 0.246 |  |
| Antiplatelet | 38 (39.2%) | 86 (29.6%) | 0.078 |  |
| Anticoagulant | 15 (15.5%) | 28 (9.6%) | 0.112 |  |
| Antihypertensive | 84 (86.6%) | 224 (77.0%) | 0.043 |  |
| ERA | 23 (23.7%) | 52 (17.9%) | 0.207 |  |
| PDEI | **15 (15.5%)** | **14 (4.8%)** | **0.001** |  |

**Supplementary Data S1. Australian Scleroderma Interest Group (ASIG) contributors**

| Maryam | Tabesh | Department of Rheumatology, St Vincent's Hospital, Melbourne, Victoria, Australia |
| --- | --- | --- |
| Jennifer | Ng | Department of Rheumatology, Gold Coast University Hospital, Southport, Australia |
| Kelly | Hollis | Department of Rheumatology, Gold Coast University Hospital, Southport, Australia |
| JOANNE | SAHHAR | Department of Rheumatology, Monash Medical Centre, Melbourne, Victoria, Australia |
| Linda | Bradbury | Department of Rheumatology, Gold Coast University Hospital, Southport, Australia |
| Lauren | Host | Fiona Stanley Hospital, Perth, Western Australia, Australia |
| Kim | Griggs | Department of Rheumatology, Royal Adelaide Hospital, Adelaide, SA, Australia |
| Leah | McWilliams |  |
| Jennifer | Walker | Department of Rheumatology, Royal Adelaide Hospital, Adelaide, SA, Australia |
| Nava | Ferdowsi |  |
| Gene-Siew | Ngian | Department of Rheumatology, Monash Medical Centre, Melbourne, Victoria, Australia |
| Emily | Lin | Department of Rheumatology, Monash Medical Centre, Melbourne, Victoria, Australia |
| Diane | Apostolopoulos | Department of Rheumatology, Monash Medical Centre, Melbourne, Victoria, Australia |
| Matthew | Parker |  |
| Peter | Youssef |  |
| Madana | Nikpour | Department of Rheumatology, Royal Prince Alfred Hospital, Camperdown, New South Wales, Australia |
| Stephanie | Frade | Sydney School of Public Health, Faculty of Medicine and Health, The University of Sydney, New South Wales, Australia |
| Thomas | Hibbert | Department of Rheumatology, Royal Prince Alfred Hospital, Camperdown, New South Wales, Australia |
| Helen | Cooley |  |
| Gabor | Major |  |
| Gemma | Strickland |  |
| Lindsey | Bunce |  |
| Helen | Marsden |  |
| Shereen | Paramalingam | Fiona Stanley Hospital, Perth, Western Australia, Australia |
| Alannah | Quinlivan |  |
| Laura | Ross | Department of Medicine, The University of Melbourne, Australia |
| Peter | Wong | Faculty of Medicine and Health, The University of Sydney, New South Wales, Australia |
| Mathuja | Bavanendrakumar |  |
| Amanda | Saracino | Department of Medicine, The University of Melbourne, Australia |
| Dylan | Hansen | Department of Rheumatology, St Vincent's Hospital, Melbourne, Victoria, Australia |
| Jessica | Fairley | Department of Rheumatology, St Vincent's Hospital, Melbourne, Victoria, Australia |
| Kimti | Kumar | Discipline of Medicine, The University of Adelaide, Adelaide, SA, Australia |
| Lucy | Croyle |  |
| Mahin | Moghaddami |  |
| Ross | Penglase |  |
| Tamera | Corte | Faculty of Medicine and Health, The University of Sydney, New South Wales, Australia |
| Tony | Kenna | Centre for Immunology and Infection Control, School of Biomedical Sciences, Queensland University of Technology, Brisbane, Queensland, Australia |
| Zoe | Brown | Department of Rheumatology, St Vincent's Hospital, Melbourne, Victoria, Australia |
| Wendy | Stevens | Department of Rheumatology, St Vincent's Hospital, Melbourne, Victoria, Australia |
| Susanna | Proudman | Department of Rheumatology, Royal Adelaide Hospital, Adelaide, SA, Australia |
